# Safety and Efficacy of Elective Switch from Nilotinib to Imatinib in Newly Diagnosed Chronic Phase Chronic Myeloid Leukemia

**DOI:** 10.1007/s44228-022-00001-x

**Published:** 2022-05-12

**Authors:** Ali Ibrahim, Nour Moukalled, Rami Mahfouz, Jean El Cheikh, Ali Bazarbachi, Iman Abou Dalle

**Affiliations:** 1 Hematology-Oncology Division, Internal Medicine Department, American University of Beirut, Beirut, Lebanon; 2 Department of Hematology and Medical Oncology, Winship Cancer Institute, Emory University, Atlanta, GA USA; 3 Department of Molecular Pathology, American University of Beirut, Beirut, Lebanon

**Keywords:** CML, Chronic phase, Imatinib, Nilotinib, Cytogenetic response, Maintenance therapy

## Abstract

The treatment of newly diagnosed chronic phase chronic myeloid leukemia (CML) with nilotinib has resulted in a higher rate of major molecular (MMR) and complete cytogenetic response (CCyR) at 12 months compared to imatinib but at a higher cumulative cost and increased risk of serious adverse events. To maintain long-term efficacy and minimize both toxicity and costs, we aimed at evaluating in a prospective single-center trial the efficacy and safety of a response-directed switch from nilotinib to imatinib after 12 months in patients newly diagnosed with chronic phase CML. Thirteen adult patients were enrolled. Twelve patients started on nilotinib 300 mg twice daily. Eleven patients completed one year of nilotinib and were switched to imatinib 400 mg daily as per protocol. At 3 months, all patients achieved a complete hematologic response, with 7 (58%) patients had early molecular response. At 12 months, all patients achieved CCyR, of whom 5 (42%) and 4 (33%) patients achieved MMR and MR4.5, respectively. Three (27%) patients switched back to nilotinib after 18, 24, and 51 months respectively: 1 patient because of loss of CCyR after 18 months, and 2 patients because of imatinib intolerance. At last follow-up, all patients (*n* = 12) were alive and in MMR, 6 (50%) of them in continuous MR4.5. These findings suggest that response directed switch from nilotinib to imatinib at 12 months is capable of maintaining long-term response, with manageable side effects. This approach warrants further exploration with larger prospective trials. Clinical trial registration: Clinicaltrials.gov identifier: NCT01316250, https://clinicaltrials.gov/ct2/results?cond=&term=NCT01316250&cntry=&state=&city=&dist=.

## Introduction

The therapeutic landscape of chronic myeloid leukemia (CML) had dramatically changed after the introduction of tyrosine kinase inhibitors (TKIs). Currently, patients with CML in chronic phase (CML-CP) have a normal life expectancy compared to age-matched healthy individuals, especially if complete cytogenetic response (CCyR) or deeper is achieved within one year of treatment [1]. More recently, the ultimate goal of CML treatment is increasingly focused on minimizing long-term toxicities, and maximizing deeper remissions in order to achieve treatment-free remissions [2]. There are multiple factors that can influence the selection of frontline treatment in CML-CP including disease-related risks, TKIs safety profile, preexisting comorbidities, and treatment goals [3]. Second generation TKIs such as nilotinib demonstrated a higher efficacy compared to imatinib in terms of achieving early molecular responses (EMR), defined as BCR-ABL1 ≤ 10% at 3 months with a rate of 91% versus 67% with imatinib, and 5-year MR4.5 rates (55% versus 35%) [4, 5]. Achieving EMR at 3 and 6 months are associated with better long-term outcomes [6]. However, long-term toxicities related to nilotinib is of significant concern, particularly cardiovascular events that occurred in about 20% of patients over a 10-year period compared with 5% with imatinib [7]. Another factor that may affect the choice of frontline TKI treatment especially in developing countries is the cumulative TKI costs representing a substantial economic burden. To date, generic imatinib is considered the most cost-effective drug among other TKIs with incorporation of treatment discontinuation strategies [8]. In order to have a balance between efficacy and safety/limiting costs, starting patients on imatinib with an early switch to second generation TKI in case of suboptimal response may seem to be beneficial [9–11]. However, in this prospective phase 2 trial, patients with newly diagnosed CML-CP were initially treated with nilotinib for 12 months duration to achieve early deeper responses, then electively switched to imatinib. The purpose of the study was to evaluate the effectiveness and safety of such strategy.

## Design and Methods

### Study Design and Participants

This is a single arm interventional phase 2 clinical trial evaluating the efficacy and safety of elective switch from nilotinib to imatinib in newly diagnosed CML-CP. Adult patients (≥ 18 years of age) with previously untreated Philadelphia chromosome-positive CML-CP, with a good Eastern Cooperative Oncology Group (ECOG) performance status (PS: 0–2), adequate hepatic and renal functions were eligible for the trial. Chronic phase CML was defined by the presence of less than 15% blasts in peripheral blood (PB) and bone marrow (BM), less than 30% blasts plus promyelocytes in PB and BM, less than 20% basophils in PB, and without extramedullary involvement, with the exception of hepatosplenomegaly. Patients were excluded if they had histopathologically confirmed central nervous system (CNS) disease, had history of cardiac dysfunction or arrhythmias, had a myocardial infarction or an unstable angina within 12 months prior to study entry, or any other clinically significant heart disease such as congestive heart failure, or uncontrolled hypertension. Exclusion criteria also included the use of therapeutic coumarin derivatives, concurrent severe and/or uncontrolled medical conditions, known HIV infection, patients with active malignancy that needs intervention, patients who are pregnant or breast feeding, or adults of childbearing potential not using an effective method of birth control.

All patients were enrolled consecutively and gave written informed consent. The study was performed in accordance with the Declaration of Helsinki. The trial design was reviewed and approved by the Institutional Review Board and was registered at ClinicalTrials.gov.

### Treatment Plan

Patients were treated with nilotinib at the standard dose of 300 mg orally twice daily for 12 months, and who achieved complete cytogenetic response (CCyR) or partial cytogenetic response (PCyR) were shifted to imatinib at a dose of 400 mg orally once daily, with regular follow ups at 3, 6, 12 months, then every 6 months thereafter.

### Clinical Endpoints

The primary endpoint was to evaluate the efficacy of imatinib in maintaining cytogenetic and molecular responses achieved at 12 months after nilotinib treatment. We anticipate that imatinib would be able to maintain cytogenetic response in 85% of patients. The assumption is based on the IRIS trial where 15.9% of patients who had a confirmed complete cytogenetic response at any time on imatinib treatment no longer had that response during longer follow-up [12]. Secondary end-points were to assess the safety of such strategy.

### Definition of Response and Response Monitoring

Response assessment were performed at 3, 6, 9, and 12 months then every 6 months thereafter for all patients as per standard practice using standard karyotype for cytogenetic analysis, and BCR-ABL1 polymerase chain reaction (PCR) to assess molecular response. Complete hematologic response (CHR) was defined by normalization of blood counts with a white cell count < 10,000/mm^3^, a platelet count < 450,000/mm^3^, < 5% myelocytes plus metamyelocytes, < 20% basophils and the absence of blasts and promyelocytes in PB, and the absence of extramedullary involvement. Cytogenetic responses were classified by standard criteria with CCyR defined as absence of Ph‐positive metaphases. A major molecular response (MMR) was defined as *BCR‐ABL1/ABL1* transcript ratio ≤ 0.1% on international scale (IS), and MR4.5 as a ratio of ≤ 0.0032% IS.

## Results

### Patients’ Characteristics

Thirteen adult patients with previously untreated CML-CP were enrolled. One patient withdrew consent before treatment initiation, thus the remaining 12 patients were evaluable for response with a median follow-up period of 54 months (range 21–87). Baseline characteristics are summarized in Table 1. The median age at diagnosis was 51 years (range 31–85), and 58% of patients had an intermediate or high sokal score. All patients started on nilotinib 300 mg twice daily after a median of 9 days (range 2–32) from diagnosis. Eleven patients completed one year of nilotinib and were switched to imatinib 400 mg daily as per protocol. One patient discontinued nilotinib after 4 months of therapy and switched to dasatinib 100 mg daily due to recurrent pancreatitis.

**Table 1:** Baseline characteristics

Variable	*N* (%)—median (range)
No. evaluable patients	12
Male gender	8 (67)
ECOG performance status ≤ 2	1 (0–2)
Sokal risk score	
Low	5 (42)
Intermediate	5 (42)
High	2 (16)
Smoking status	5 (42)
Age—years	51 (31–85)
WBCs—/mm^3^	91,750 (19,500–464,000)
Platelets—/mm^3^	284,000 (152,000–794,000)
Median time from diagnosis to treatment, days	9 (2–32)
Median follow-up in months	54 (21–87)

### Rate of Hematologic, Cytogenetic and Molecular Responses

At 3 months, all (100%) patients achieved CHR, seven (58%) had EMR, of them three (25%) patients had MMR. At 6 months, ten (83%) patients achieved CCyR, of whom 4 (33%) patients had MMR and 2 (17%) patients had MR4.5.

**Fig. 1:**
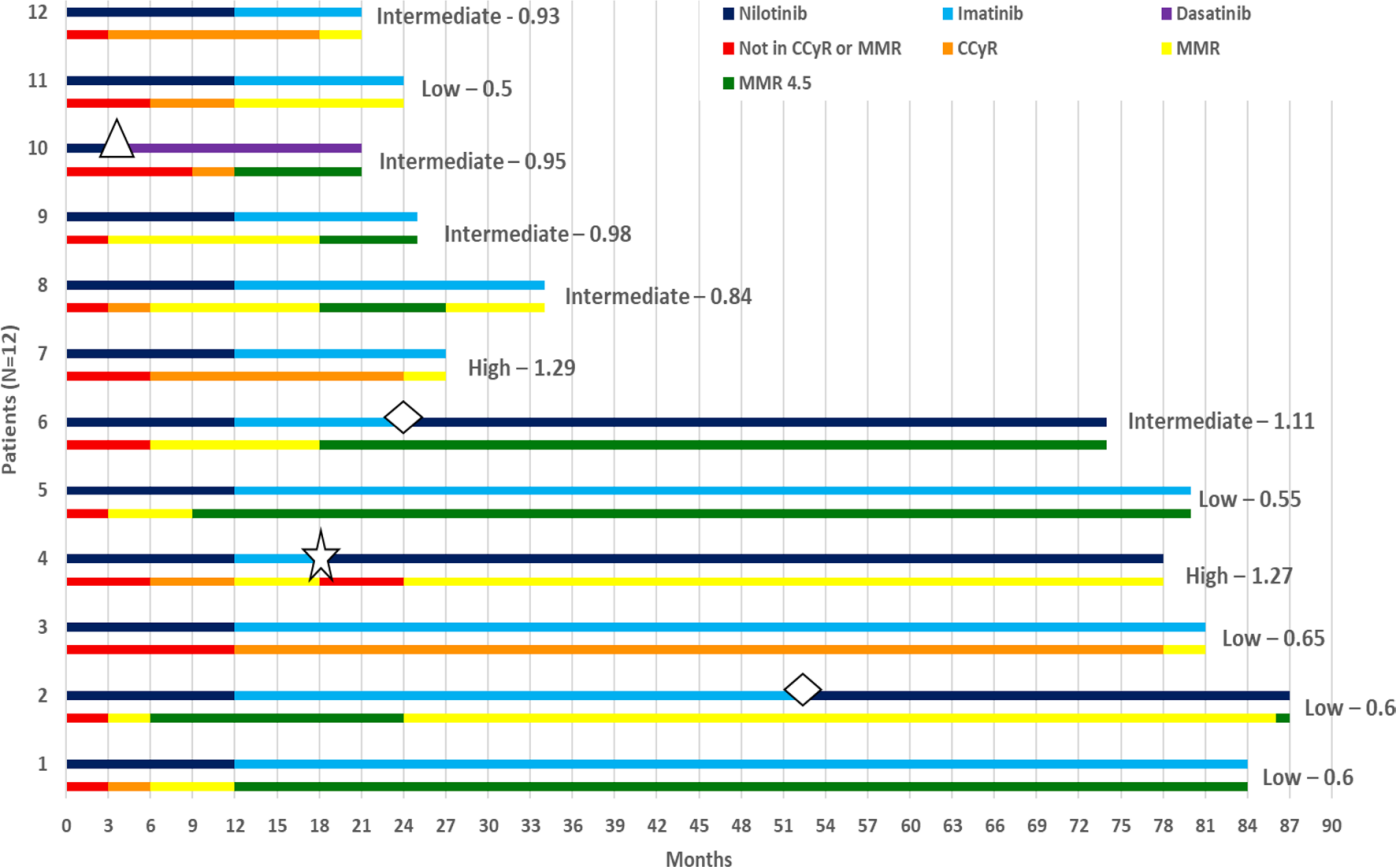
The swimmers plot illustrates the duration of treatment with each tyrosine kinase inhibitor (Upper Bar) for 12 CML patients and marks the response milestones (Lower Bar). The sokal score is indicated at the end of the horizontal bar for each patient. *Unfilled triangle* Nilotinib intolerance (Pancreatitis). *Unfilled diamond* Imatinib intolerance (Severe Myalgia). *Unfilled star* Loss of response on imatinib

At 12 months, all (100%) patients achieved CCyR, of whom 5 (42%) patients had MMR and 4 (33%) patients achieved MR4.5. At 12 months, eleven patients were switched to imatinib maintenance, of them 3 (27%) patients were switched back to nilotinib 300 mg twice daily after 18, 24, 51 months respectively: one patient because of loss of CCyR (BCR-ABL: 0.11% IS) on imatinib after 18 months, and two patients because of imatinib intolerance (grade 3 myalgias). At 18 months, 11 (92%) patients were in CCyR, nine (75%) patients were in MMR, of them 6 (50%) patients were in MR4.5, only one patient lost CCyR. At last follow up, 12 patients (100%) were alive and in CCyR and MMR, 6 (50%) of them in continuous MR4.5, none of the patients attempted treatment discontinuation or developed long-term grade 3–4 adverse events, such as cardiovascular or metabolic complications. There was no progression to accelerated or blast phase CML. After a median follow up of 54 months (range 21–87), eight patients remained on imatinib without evidence of progression. The three patients who were switched back to nilotinib remained on nilotinib 300 mg twice daily without signs of progression. The patient who lost CCyR after 6 months of switching to imatinib, regained a MMR after 6 months of resuming nilotinib, and continued in MMR at last follow-up (Fig. 1).

The median time to CHR, MMR, MR 4.5, and CCyR were 3 months (range 1–3), 6 months (range 3–24), 12 months (range 6–24), and 4.5 months (range 3–12) respectively.

### Safety

One patient had to switch to dasatinib after 4 months of treatment with nilotinib because of symptomatic recurrent pancreatitis. Two patients on imatinib switched back to nilotinib because of imatinib related grade 3 severe myalgias.

## Discussion

A major revolution in the treatment of CML was the advent of TKIs that transformed CML from a disease with limited therapeutic options to a chronic disease. While in the past the primary goal of the treatment was to achieve response and prolong survival, currently, the overall survival of CML patients approaches the age and sex-matched general population life expectancy, and the need for ongoing TKI therapy is now controversial, knowing the cumulative cost and long-term toxicity. Nilotinib is a second generation TKI that is 30-fold more potent than imatinib [13]. In the 5- and 10-years update of the randomized ENESTnd trial, nilotinib continued to show superior efficacy over imatinib [5, 7]. However, the cumulative incidence of cardiovascular events continued to increase with longer follow-up. At 5-years follow-up, 13% of patients experienced cardiovascular events in the arm of nilotinib 300 mg twice daily, compared to 3% in the imatinib control population [5]. Moreover, nilotinib is capable of achieving higher rates of early molecular response and BCR-ABL IS ≤ 1% at 3 and 6 months compared to imatinib. [5] Three- month BCR-ABL IS levels of less than 10% are predictive of long-term outcomes in CML including higher rates of MMR and MR 4.5, and a rapid initial decline in BCR-ABL IS levels also correlate with subsequent achievement of treatment free remission [4, 14].

We therefore investigated the use of frontline nilotinib aiming at achieving early molecular response and higher rate of MMR at 12 months, then switching to imatinib after 12 months of treatment with the goal of assessing its ability to maintain molecular responses, with a relatively safer long-term profile.

At 12 months, 75% of patients achieved MMR, of them 44% attained MR4.5 on nilotinib treatment. After switching to imatinib, only one patient with an initial high sokal risk score lost response after 18 months of treatment. Long-term imatinib treatment after a median follow-up of 4.5 years was well tolerated with no reported major adverse events. Only two patients discontinued imatinib early on because of non-hematologic non-cardiovascular toxicities.

The selection of frontline treatment in CML-CP should take in consideration multiple variables, including disease-related risks, patient comorbidities, costs, long-term side effects and treatment goals. The question was always addressing whether to start with a second generation TKIs with higher efficacy and higher likelihood of achieving treatment free remission, or to start with imatinib with a subsequent switch to second generation TKIs in case of suboptimal response [3]. An elective switch from a second generation TKIs to imatinib was retrospectively evaluated on 20 CML patients, and showed that imatinib can be safely and effectively administered following an optimal response at 3 months to second generation TKIs [15].

Despite the limited number of patients included in this study, we showed that a response-directed strategy can offer patients early deep remissions and high rate of 12-month CCyR that are maintained or even deepened with imatinib in 90% of the cases. The long-term imatinib treatment is likely to be safer and cost effective, especially with the introduction of generic imatinib. [16] This study serves as a pilot study for a later research to be carried out investigating alternative approaches like a response—directed switch from nilotinib to imatinib. Those strategies need further exploration by large randomized trials to find out the best balance between deep remission and long-term safety.

## Data Availability

Original data will be available upon request. Please email corresponding author on ia41@aub.edu.lb.
